# Serum Malondialdehyde-Modified Low-Density Lipoprotein Level May Be a Biomarker Associated with Aortic Stiffness Among Patients Undergoing Peritoneal Dialysis

**DOI:** 10.3390/life14111385

**Published:** 2024-10-28

**Authors:** Yu-Chi Chang, Chih-Hsien Wang, Chi-Chong Tang, Yu-Li Lin, Yu-Hsien Lai, Chiu-Huang Kuo, Bang-Gee Hsu

**Affiliations:** 1 Division of Nephrology, Hualien Tzu Chi Hospital, Buddhist Tzu Chi Medical Foundation, Hualien 97004, Taiwan; ck11516@gmail.com (Y.-C.C.); wangch33@gmail.com (C.-H.W.); dearedward1025@gmail.com (C.-C.T.); nomo8931126@gmail.com (Y.-L.L.); hsienhsien@gmail.com (Y.-H.L.); hermit.kuo@gmail.com (C.-H.K.); 2 School of Medicine, Tzu Chi University, Hualien 97004, Taiwan; 3 Institute of Medical Sciences, Tzu Chi University, Hualien 97004, Taiwan; 4 School of Post-Baccalaureate Chinese Medicine, Tzu Chi University, Hualien 97004, Taiwan

**Keywords:** malondialdehyde-oxidized low-density lipoprotein, carotid–femoral pulse wave velocity, aortic stiffness, peritoneal dialysis

## Abstract

Background: Serum malondialdehyde-oxidized low-density lipoprotein (MDA-oxLDL) is associated with atherosclerosis and increased risk of cardiovascular disease (CVD). Vascular calcification frequently occurs with arterial stiffness in patients on peritoneal dialysis (PD). This cross-sectional study aimed to elucidate the correlation between aortic stiffness and MDA-oxLDL levels in patients on PD. Methods: Overall, 92 patients on PD were included. The carotid–femoral pulse wave velocity (cfPWV) was evaluated using cuff-based volumetric displacement, and blood samples were obtained from all patients. Aortic stiffness was classified based on cfPWV values (>10 m/s indicating aortic stiffness). Serum MDA-ox-LDL levels were quantified using commercial enzyme-linked immunosorbent assay kits. Results: In total, 33 (35.9%) patients were classified into the aortic stiffness group. Factors, including systolic blood pressure (SBP), serum triglyceride levels, C-reactive protein levels, age, weight, body mass index (BMI), waist circumference, MDA-oxLDL levels, and diabetes mellitus (DM) prevalence, were significantly higher in the aortic stiffness group. Multivariable logistic regression analysis revealed significant associations between aortic stiffness and MDA-oxLDL levels, BMI, and SBP. Furthermore, multivariable forward stepwise linear regression analysis revealed serum MDA-oxLDL levels as a significant independent predictor of cfPWV values. Conclusions: Serum MDA-oxLDL levels correlate positively with cfPWV values and may predict aortic stiffness among PD patients, highlighting its potential role in assessing CVD risk in this population.

## 1. Introduction

Cardiovascular disease (CVD) is the primary cause of mortality and hospitalization among individuals on peritoneal dialysis (PD) [1,2,3]. Vascular calcifications and increased arterial stiffness are significant cardiovascular risk factors in these patients [2], underscoring the need for the timely identification of high-risk individuals and effective primary prevention strategies. Arterial stiffness, which is a predictor of CVD and mortality, can be detected early before the onset of elevated blood pressure [4]. The pulse wave velocity (PWV), which is measured between the carotid and femoral arteries (cfPWV), is the gold standard for evaluating aortic stiffness [5,6].

Patients with PD exhibit unique atherogenic lipid profiles beyond classic dyslipidemia that are attributable to elevated glucose levels and protein losses associated with dialysate exposure, thereby contributing to the progression of atherosclerosis and chronic inflammation [7,8]. Lipid metabolism aberrations in patients with PD correlate with an increased risk of technique failure [9], as well as the development and progression of arterial stiffness [10], cardiovascular events, and all-cause mortality [11,12]. Oxidized low-density lipoprotein (ox-LDL) stimulates collagen fiber formation in vascular smooth muscle cells (VSMCs), accelerates intimal thickening, and attenuates the biochemical effects of nitric oxide (NO), all of which are implicated in the development and progression of arterial stiffness [13]. Serum malondialdehyde-oxidized LDL (MDA-oxLDL) is an ox-LDL with apolipoprotein B bound to malondialdehyde (MDA). MDA is one of the key aldehydes formed during oxidative stress and its modification of LDL is often used as a marker for oxidative damage and inflammation. When LDL is oxidized, it becomes more atherogenic, meaning it is more likely to contribute to the formation of plaque in arteries, which can play a crucial role in atherosclerosis development and has been linked to major adverse cardiovascular events [14,15].

The relationship between serum MDA-oxLDL levels and aortic stiffness in patients with PD remains unclear. This study aimed to explore the relationship between aortic stiffness and serum MDA-oxLDL levels by assessing cfPWV values in patients with PD.

## 2. Materials and Methods

### 2.1. Participants

Overall, 92 participants who maintained PD for >3 months at Hualien Tzu Chi Hospital between January 2021 and October 2021 were enrolled in this cross-sectional study. Systolic (SBP) and diastolic (DBP) blood pressures were evaluated in the morning after at least 10 min of rest using standard mercury sphygmomanometers and appropriately sized cuffs. BP readings were obtained three times at 5 min intervals. Diabetes mellitus (DM) was defined as a fasting serum glucose level of ≥126 mg/dL or the use of oral hypoglycemic agents or insulin. Hypertension (HTN) was defined as an SBP of ≥140 mmHg and a DBP of ≥90 mmHg or the use of any antihypertensive medication within the preceding 2 weeks. In total, 60 patients underwent continuous ambulatory PD (CAPD) with 3–5 daily dialysate exchanges, while 32 patients received 3–5 nightly exchanges using an automated PD (APD) device. Dialysis adequacy was evaluated by reviewing medical records, including total creatinine clearance, the weekly fractional clearance index for urea (weekly Kt/V), and peritoneal creatinine clearance. The study protocol was approved by the Buddhist Tzu Chi Medical Foundation’s research ethics committee of Hualien Tzu Chi Hospital (IRB108-219-A). Informed consent was obtained from all participants before the study commencement. Exclusion criteria included patients who refused to provide informed consent and those with acute illnesses at the time of blood sampling, including acute myocardial infarction, acute infection, amputation, heart failure, malignancy, or pulmonary edema.

### 2.2. Anthropometric Analyses

Anthropometric measurements were obtained, including height, weight, and waist circumference. Waist circumference was measured at the midpoint between the lowest ribs and the iliac crest with the hands on the hips. Body mass index (BMI) was calculated as weight (in kilograms) divided by the square of height (in meters). All measurements were rounded to the nearest 0.5 cm and 0.5 kg, with participants wearing lightweight clothing and no shoes [16,17].

### 2.3. Analyses of Biochemistry

Biochemical analyses were performed on fasting blood samples obtained from each patient, centrifuged at 3000× *g* for 10 min at 4 °C, and analyzed within 1 h of collection. Serum concentrations of albumin, blood urea nitrogen (BUN), creatinine, C-reactive protein (CRP), fasting glucose, phosphorus, total calcium, total cholesterol (TCH), and triglycerides (TG) were measured using an autoanalyzer (Siemens Advia 1800, Siemens Healthcare GmbH, Henkestr, Germany) [16,17]. Commercially available enzyme-linked immunosorbent assays were used to quantify serum levels of MDA-oxLDL (Biomedica Divischgasse, Vienna, Austria) and intact parathyroid hormone (iPTH) (IBL International GmbH, Hamburg, Germany).

### 2.4. Measurement of cfPWV

To assess AS, cfPWV measurements were conducted using cuff-based volumetric displacement (SphygmoCor XCEL, AtCor Medical, Sydney, NSW, Australia) as previously described [17,18]. Participants rested for 10 min before measurements were obtained in the morning while lying supine in a quiet, temperature-controlled environment. The XCEL device’s cuff was placed on the left upper arm. Brachial SBP and DBP were automatically recorded using standard oscillometric measurements followed by immediate reinflation of the cuff to a subdiastolic pressure level. The XCEL system substitutes the volumetric displacement waveform from an upper-thigh cuff for femoral artery tonometry to measure the cfPWV. Meanwhile, tonometry measures the carotid pulse [17,18]. This study defined the high aortic stiffness group as patients with cfPWV values > 10 m/s, whereas those with cfPWV values ≤ 10 m/s were classified as the control group, following the guidelines from the European Society of Hypertension and the European Society of Cardiology [19].

### 2.5. Statistical Analyses

To detect a correlation coefficient of about 0.3 between the serum MDA-oxLDL levels and cfPWV, with an alpha level of 0.05 and a power of 80%, a total of at least 85 patients should be included in this study.

All statistical analyses were conducted using SPSS for Windows (version 19.0, IBM Corp., Armonk, NY, USA). The Kolmogorov–Smirnov test assessed normal distribution in continuous variables, whereas those not conforming to normal distribution were assessed using logarithmic transformation for inclusion in linear regression analyses. Normally distributed continuous variables are presented as mean ± standard deviation and between-group comparisons were performed using Student’s independent *t*-tests (two-tailed). Non-normally distributed variables are presented as medians and interquartile ranges, and between-group comparisons were made using the Mann–Whitney U test (CRP, fasting glucose, iPTH, MDA-oxLDL, PD vintage, TG, and weekly Kt/V). Categorical variables are presented as numbers and percentages and were evaluated using the χ^2^ test. Multivariate logistic regression analysis was conducted to examine the risk factors related to the progression of aortic stiffness among patients with PD. Univariate and multivariate forward stepwise linear regression analyses assessed the relationship between cfPWV and these variables. A receiver operating characteristic curve analysis was employed to calculate the area under the curve (AUC) and determine the optimal cutoff point of MDA-oxLDL levels for predicting aortic stiffness in patients with PD. All *p* values < 0.05 were deemed as statistically significant.

## 3. Results

**Figure 1:**
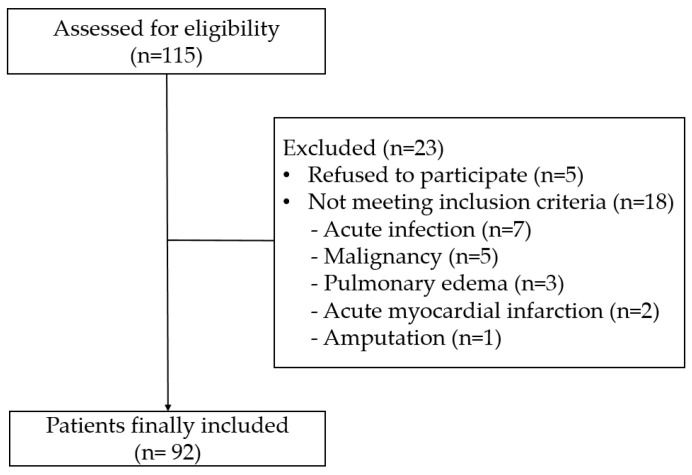
Flow chart of this study.

Out of 115 PD patients screened, 5 refused to participate, and 18 did not meet the inclusion criteria. Therefore, 92 patients were ultimately included in this study (Figure 1).

The baseline characteristics of the 92 patients in this study are shown in Table 1. The mean age of participants was 58.0 ± 14.5 years, and the median PD duration was 49 months. Overall, 95 (55.2%) patients were women, 60 (65.2%) utilized the CAPD modality, and the median total fractional clearance index for urea (Kt/V) was 1.97. Comorbidities, such as HTN, were noted in 68 patients (73.9%); DM was present in 39 patients (42.4%).

Of the 92 patients, 33 (35.9%) were included in the aortic stiffness group. Compared to the control group, patients in the aortic stiffness group were older (*p* = 0.011), had a higher prevalence of DM (*p* < 0.001), higher BMI (*p* < 0.001), greater waist circumference (*p* < 0.001), and higher SBP (*p* = 0.034), serum CRP (*p* = 0.012), TG (*p* = 0.025), and MDA-oxLDL (*p* = 0.001) levels.

Table 2 shows the results of a multivariate logistic regression analysis of factors correlated with aortic stiffness among patients with PD. In this study, increased serum MDA-oxLDL levels (odds ratio [OR], 1.171; 95% confidence interval [CI], 1.021–1.342; *p* = 0.024]), an elevated BMI (OR, 1.965; 95% CI, 1.270–3.038; *p* = 0.002), higher SBP (OR, 1.040; 95% CI, 1.006–1.074; *p* = 0.020), and presence of DM (OR, 7.685; 95% CI, 1.879–31.436; *p* = 0.005) were independent predictors of aortic stiffness progression among patients with PD.

Table 3 shows the correlation between cfPWV, serum log-transformed MDA-oxLDL (log-MDA-oxLDL) levels, and clinical variables. cfPWV positively correlated with age (*r* = 0.350, *p* = 0.001), BMI (*r* = 0.437, *p* < 0.001), SBP (*r* = 0.376, *p* < 0.001), logarithmically transformed TG levels (log-TG, *r* = 0.230, *p* = 0.028), log-CRP levels (*r* = 0.318, *p* = 0.002), and log-MDA-oxLDL levels (*r* = 0.469, *p* < 0.001). After adjusting for significant covariates (age, BMI, SBP, log-TG, log-CRP, and log-MDA-oxLDL), multivariable forward stepwise linear regression analysis showed that age (β = 0.201, adjusted R^2^ change = 0.028, *p* = 0.023), BMI (β = 0.236, adjusted R^2^ change = 0.057, *p* = 0.006), SBP (β = 0.246, adjusted R^2^ change = 0.035, *p* = 0.004), and serum log-MDA-oxLDL levels (β = 0.253, adjusted R^2^ change = 0.212, *p* = 0.005) were independently associated with cfPWV.

**Figure 2:**
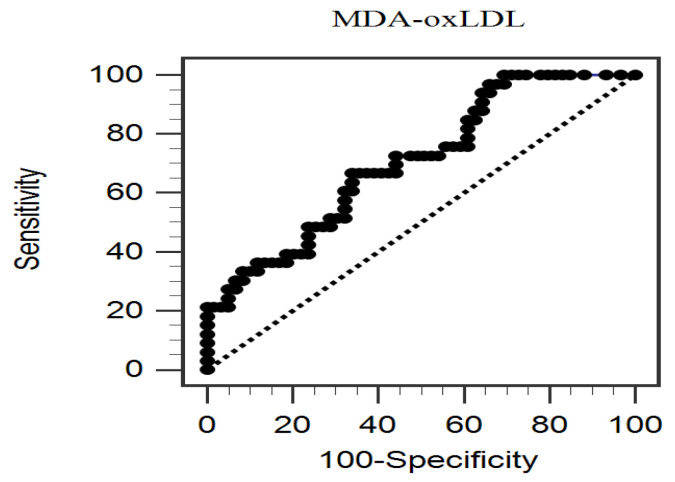
The area under the receiver operating characteristic curve shows the diagnostic power of malondialdehyde-oxidized low-density lipoprotein levels for predicting aortic stiffness among 92 patients with PD.

Figure 2 presents the ideal cutoff level of serum MDA-oxLDL (1.73 μg/mL) for predicting aortic stiffness, as determined by the AUC (0.705; 95% CI, 0.597–0.813; *p* = 0.0002). This level had a sensitivity of 66.7%, specificity of 66.1%, positive predictive value of 52.4%, and negative predictive value of 78.0%.

## 4. Discussion

This study showed that BMI, SBP, DM, and serum MDA-oxLDL levels may independently predict aortic stiffness in patients with PD. After adjusting for confounding covariates, multivariable forward stepwise linear regression analysis revealed that age, BMI, SBP, DM, and serum log-MDA-oxLDL levels were associated with cfPWV.

Previous studies have demonstrated that advanced age is a significant risk factor in the progression of arterial stiffness and the subsequent development of CVD [18,20,21]. Age-related changes in the aorta and central arteries result in a loss of elasticity that often coincides with an elevated SBP and pulse rates and a decline in the DBP [20,22]. Patients on PD have significant aortic stiffness compared to those on hemodialysis, with a statistically significant association between cfPWV and age [23]. These findings are consistent with and support the results of our study.

Obesity and metabolic syndrome have been implicated in the development of aortic stiffness and CVD in the general population [22,24]; this phenomenon is also observed among patients with PD [2,7,25]. In the general population, Kim et al. reported that the brachial–ankle PWV (baPWV) was associated with the visceral fat area and waist–hip ratio, but not with the BMI and waist circumference [26]. This indicates that abdominal obesity may influence arterial stiffness more than overall obesity. Our study utilized cfPWV to evaluate aortic stiffness, representing central arterial stiffness. Our findings showed that the BMI is an independent predictor, and waist circumference is a significant predictor of aortic stiffness in patients with PD. The arterial stiffness assessment is performed by measuring the cfPWV, which is the gold standard [5,6].

Our previous study investigated glucometabolic indices in patients with PD, revealing significant correlations between impaired fasting glucose, insulin resistance, the PD glucose load, and aortic stiffness, particularly in patients without diabetes on PD [27]. Furthermore, among patients with PD, those with DM had the highest levels of aortic stiffness compared to those without DM [27]. Moreover, Hu et al. highlighted PD as a risk factor for both cardiac and all-cause mortality in patients on dialysis, particularly among those with DM [28]. Similarly, our study revealed an association between DM and aortic stiffness among patients with PD, underscoring the increased cardiovascular risk faced by patients with DM on PD.

The relationship between CRP and cardiovascular outcomes and mortality among patients with PD has been established [29,30]. CRP, an inflammation marker, correlates with aortic stiffness as measured by cfPWV [31]. This correlation is supported by several potential mechanisms, including decreased NO bioavailability and production by endothelial cells resulting in endothelial dysfunction [32], increased matrix metalloproteinase activity from leukocytes leading to elastin fiber degradation within the arterial wall [33], increased bioapatite production from VSMCs contributing to arterial wall calcification [34], and the upregulation of glycosaminoglycans (such as decorin, versican, biglycan, and hyaluronan) that reinforce arterial stiffness [35,36]. However, contrary to our results, serum CRP levels were not significantly associated with aortic stiffness (*p* = 0.116) after adjusting for confounding factors in multivariate logistic regression analysis.

Lipid metabolism derangement is observed in patients with PD, contributing to aortic stiffness, CVD, and mortality [7,12,24,37]. Additionally, longitudinal prospective studies have emphasized the association between serum TG levels and PWV. Wang et al. discovered a positive relationship between TG levels and cfPWV during the follow-up period, indicating that reducing TG levels could be a potential treatment strategy for patients with CVD [38]. Similarly, in another study, baseline serum TG levels independently predicted the progression of arterial stiffness in healthy men even after adjusting for confounding factors and variables [39]. Similar to our findings, TG levels exhibited a near-significant association with aortic stiffness, whereas TCH levels did not.

MDA, a byproduct of lipid peroxidation, is associated with baPWV and is a surrogate marker for oxidative stress [10]. Kim et al. showed that serum MDA-LDL and subsequent ox-LDL production exert direct cytotoxic effects on endothelial cells, trigger the exocytosis of adhesion molecules, induce platelet aggregation and monocyte adhesion, and contribute to foam cell formation within atherosclerotic lesions, collectively influencing baPWV and inducing arterial wall remodeling [40]. Elevated serum MDA levels are associated with adverse effects on aortic stiffness in populations at high cardiovascular risk. Our previous study noted that the serum MDA-oxLDL level is associated with arterial stiffness measured by the cardio-ankle vascular index in patients with triple-vessel coronary artery disease who underwent coronary artery bypass graft (CABG) surgery [21]. In our study, the serum MDA-oxLDL levels positively correlated with cfPWV values in patients with PD, which parallels those of a cross-sectional study involving patients with regular hemodialysis [41]. Despite patients on PD having a high burden of vascular calcification and exposure to glucose-containing dialysate, our study underscores the predictive value of serum MDA-oxLDL in detecting aortic stiffness in these patients.

This study has several limitations. First, its observational design and enrollment from a single center with cross-sectional analysis prevent the establishment of causality. Second, some results’ lack of statistical significance may be due to the relatively small sample size. Third, patients with PD are a minority within the end-stage renal disease population, potentially limiting the generalizability of our findings regarding the correlation between MDA-oxLDL and aortic stiffness in patients with PD. Fourth, while our study emphasizes the positive association between serum MDA-oxLDL levels and cfPWV values, further research is warranted to fully understand the comprehensive mechanisms and roles of MDA-oxLDL in the development and progression of aortic stiffness.

## 5. Conclusions

Our findings suggest a positive association between serum MDA-oxLDL levels and cfPWV values, suggesting MDA-oxLDL is an independent predictor of aortic stiffness in patients with PD. Longitudinal studies are needed to establish the causal relationship between serum MDA-oxLDL levels and the progression of atherosclerosis and arterial stiffness in PD patients. Additionally, further studies should investigate whether lowering serum MDA-oxLDL levels could mitigate the progression of arterial stiffness.

## Figures and Tables

**Table 1:** Baseline characteristics of participants.

Characteristic	All Participants (*n* = 92)	Control Group (*n* = 59)	Aortic Stiffness Group (*n* = 33)	*p* Value
Age (years)	57.96 ± 14.46	55.10 ± 15.01	63.068 ± 12.02	0.011 *
Peritoneal dialysis vintage (months)	48.54 (21.03–81.42)	51.24 (22.92–80.76)	34.20 (18.12–82.68)	0.523
Body mass index (kg/m^2^)	25.11 ± 4.39	23.84 ± 3.63	27.40 ± 4.75	<0.001 *
Waist circumference (cm)	91.60 ± 11.37	88.54 ± 10.72	97.06 ± 10.56	<0.001 *
Carotid–femoral PWV (m/s)	9.58 ± 1.67	8.60 ± 0.88	11.34 ± 1.26	<0.001 *
Systolic blood pressure (mmHg)	149.46 ± 22.44	145.76 ± 21.04	156.06 ± 23.65	0.034 *
Diastolic blood pressure (mmHg)	87.76 ± 14.93	87.02 ± 15.91	89.09 ± 13.12	0.526
Total cholesterol (mg/dL)	160.29 ± 43.57	162.97 ± 47.11	155.52 ± 36.58	0.434
Triglyceride (mg/dL)	123.50 (82.00–192.00)	104.00 (76.00–167.00)	151.00 (105.00–223.00)	0.025 *
Fasting glucose (mg/dL)	112.00 (94.25–137.00)	112.00 (94.00–134.00)	112.00 (93.50–154.50)	0.858
Albumin (mg/dL)	3.53 ± 0.35	3.58 ± 0.33	3.45 ± 0.36	0.088
Blood urea nitrogen (mg/dL)	61.77 ± 21.99	63.92 ± 23.14	57.94 ± 19.52	0.213
Creatinine (mg/dL)	10.49 ± 3.39	10.94 ± 3.49	9.68 ± 3.10	0.089
Total calcium (mg/dL)	9.63 ± 0.58	9.55 ± 0.58	9.78 ± 0.55	0.060
Phosphorus (mg/dL)	5.26 ± 1.34	5.36 ± 1.42	5.08 ± 1.17	0.332
Intact parathyroid hormone (pg/mL)	184.35 (79.98–446.88)	228.10 (83.40–447.00)	160.60 (40.25–399.50)	0.227
C-reactive protein (mg/dL)	0.18 (0.11–0.94)	0.16 (0.11–0.38)	0.36 (0.14–1.88)	0.012 *
MDA-oxLDL (μg/ mL)	1.31 (0.41–6.88)	1.05 (0.18–3.68)	3.13 (0.84–12.39)	0.001 *
Weekly Kt/V	1.97 (1.71–2.17)	1.91 (1.69–2.18)	1.99 (1.77–2.14)	0.474
Peritoneal Kt/V	1.76 ± 0.39	1.77 ± 0.42	1.74 ± 0.34	0.708
Total clearance of creatinine (L/week)	58.13 ± 16.40	57.73 ± 16.35	58.84 ± 16.72	0.758
Peritoneal clearance of creatinine (L/week)	47.39 ± 12.34	47.80 ± 12.30	46.66 ± 12.58	0.674
Female, *n* (%)	51 (55.4)	35 (59.3)	16 (48.5)	0.316
Diabetes, *n* (%)	39 (42.4)	15 (25.4)	24 (72.7)	<0.001 *
Hypertension, *n* (%)	68 (73.9)	44 (74.6)	24 (72.7)	0.846
CAPD, *n* (%)	60 (65.2)	41 (69.5)	19 (57.6)	0.250
ARB use, *n* (%)	52 (56.5)	33 (55.9)	19 (57.6)	0.879
β-blocker use, *n* (%)	35 (38.0)	24 (40.7)	11 (33.3)	0.486
CCB use, *n* (%)	49 (53.3)	29 (49.2)	20 (60.6)	0.291
α-blocker use, *n* (%)	10 (10.9)	7 (11.9)	3 (9.1)	0.682
Statin use, *n* (%)	29 (31.5)	19 (32.2)	10 (30.3)	0.851
Fibrate use, *n* (%)	26 (28.3)	15 (25.4)	11 (33.3)	0.419

Values for continuous variables are shown as mean ± standard deviation after analysis by Student’s *t*-test; variables not normally distributed are shown as median and interquartile range after analysis by the Mann–Whitney U test; values are presented as number (%) and analysis after analysis by the Chi-square test. Malondialdehyde-oxidized low-density lipoprotein, MDA-oxLDL; CAPD, continuous ambulatory peritoneal dialysis; Weekly Kt/V, weekly fractional clearance index for urea; ARB, angiotensin-receptor blocker; CCB, calcium-channel blocker. * *p* < 0.05 was considered statistically significant.

**Table 2:** Multivariate logistic regression analysis of the factors correlated with aortic stiffness.

Variables	Odds Ratio	95% Confidence Interval	*p* Value
MDA-oxLDL, 1 μg/ mL	1.171	1.021–1.342	0.024 *
Diabetes, present	7.685	1.879–31.436	0.005 *
Body mass index, 1 kg/m^2^	1.965	1.270–3.038	0.002 *
Systolic blood pressure, 1 mmHg	1.040	1.006–1.074	0.020 *
Age, 1 year	1.061	0.997–1.129	0.062
Waist circumference, 1 cm	0.864	0.746–1.001	0.051
Triglyceride, 1 mg/dL	1.005	0.999–1.010	0.084
C-reactive protein, 1 mg/dL	1.623	0.887–2.971	0.116

Data were analyzed using the multivariate logistic regression analysis (adopted factors: diabetes, body mass index, waist circumference, age, systolic blood pressure, triglyceride, C-reactive protein, and MDA-oxLDL). Malondialdehyde-oxidized low-density lipoprotein, MDA-oxLDL. * *p* < 0.05 was considered statistically significant.

**Table 3:** Correlation between carotid–femoral pulse wave velocity levels and clinical variables.

Variables	Carotid–Femoral Pulse Wave Velocity (m/s)				
	Uni-Variable Regression		Multi-Variable Regression		
	*r*	*p* Value	Beta	Adjusted R^2^	*p* Value
Age (years)	0.350	0.001 *	0.201	0.028	0.023 *
Log-PD vintage (months)	0.044	0.678	–	–	–
Body mass index (kg/m^2^)	0.437	<0.001 *	0.236	0.057	0.006 *
Waist circumference (cm)	0.258	0.013 *			
Systolic blood pressure (mmHg)	0.376	<0.001 *	0.246	0.035	0.004 *
Diastolic blood pressure (mmHg)	0.070	0.506	–	–	–
Total cholesterol (mg/dl)	−0.079	0.456	–	–	–
Log-Triglyceride (mg/dL)	0.230	0.028 *	–	–	–
Log-Glucose (mg/dL)	0.062	0.556	–	–	–
Albumin (mg/dL)	−0.116	0.271	–	–	–
Blood urea nitrogen (mg/dL)	−0.104	0.324	–	–	–
Creatinine (mg/dL)	−0.006	0.952	–	–	–
Total calcium (mg/dL)	0.131	0.214	–	–	–
Phosphorus (mg/dL)	−0.092	0.382	–	–	–
Log-iPTH (pg/mL)	−0.157	0.136	–	–	–
Log-CRP (mg/dL)	0.318	0.002 *	–	–	–
Log-MDA-oxLDL (μg/ mL)	0.469	<0.001 *	0.253	0.212	0.005 *
Log-Weekly Kt/V	0.069	0.515	–	–	–
Peritoneal Kt/V	0.050	0.635	–	–	–
Total clearance of creatinine (L/week)	−0.004	0.973	–	–	–
Peritoneal clearance of creatinine (L/week)	−0.013	0.905	–	–	–

Data of PD vintage, triglyceride, glucose, iPTH, CRP, Weekly Kt/V, and MDA-oxLDL levels showed skewed distribution and were log-transformed before analysis. Analysis data were completed using the simple regression analyses or multivariate stepwise linear regression analysis (adopted factors: age, body weight, body mass index, systolic blood pressure, log-triglyceride, log-CRP, and log-MDA-oxLDL). PD, peritoneal dialysis; iPTH, Intact parathyroid hormone; CRP, C-reactive protein; Weekly Kt/V, weekly fractional clearance index for urea; Malondialdehyde-oxidized low-density lipoprotein, MDA-oxLDL. * *p* < 0.05 was considered statistically significant.

## Data Availability

The raw data supporting the conclusions of this article will be made available by the authors on request.
